# A New Approach in Regression Analysis for Modeling Adsorption Isotherms

**DOI:** 10.1155/2014/930879

**Published:** 2014-01-30

**Authors:** Dana D. Marković, Branislava M. Lekić, Vladana N. Rajaković-Ognjanović, Antonije E. Onjia, Ljubinka V. Rajaković

**Affiliations:** ^1^Faculty of Technology and Metallurgy, University of Belgrade, Karnegijeva 4, 11000 Belgrade, Serbia; ^2^Faculty of Civil Engineering, University of Belgrade, Bulevar Kralja Aleksandra 73, 11000 Belgrade, Serbia; ^3^Vinča Institute of Nuclear Sciences, University of Belgrade, P.O. Box 522, 11001 Belgrade, Serbia

## Abstract

Numerous regression approaches to isotherm parameters estimation appear in the literature. The real insight into the proper modeling pattern can be achieved only by testing methods on a very big number of cases. Experimentally, it cannot be done in a reasonable time, so the Monte Carlo simulation method was applied. The objective of this paper is to introduce and compare numerical approaches that involve different levels of knowledge about the noise structure of the analytical method used for initial and equilibrium concentration determination. Six levels of homoscedastic noise and five types of heteroscedastic noise precision models were considered. Performance of the methods was statistically evaluated based on median percentage error and mean absolute relative error in parameter estimates. The present study showed a clear distinction between two cases. When equilibrium experiments are performed only once, for the homoscedastic case, the winning error function is ordinary least squares, while for the case of heteroscedastic noise the use of orthogonal distance regression or Margart's percent standard deviation is suggested. It was found that in case when experiments are repeated three times the simple method of weighted least squares performed as well as more complicated orthogonal distance regression method.

## 1. Introduction

Adsorption is a mass transfer process that plays a central role in potable water purification, wastewater treatment, both analytical and preparative chromatograph, and different types of chemical analyses as a technique for sample preconcentration and speciation of analytes. The predominant scientific basis for sorbent selection and design of an adsorption system is the knowledge about equilibrium partitioning between two phases often expressed in the form of adsorption isotherm. Based on the isotherms, the following important factors can be estimated: capacity of the sorbent, the method of sorbent regeneration, and the product purities [1]. Additionally, transport behavior of environmentally significant reactive species is controlled by the sorption behavior of these solutes to soil surfaces. Adsorption isotherms are incorporated into geochemical modeling software (such as MINEQL, Visual MINTEQ, and ChemEQL) in order to understand and predict the mobility of the sorbed substances. Determination of the optimum isotherm equation and accurate estimates of the isotherm parameters are apparently important for all the mentioned purposes.

A frequently applied method for determining single solute adsorption isotherms is the conventional batch method based on mixing known amounts of adsorbent with solutions of various initial concentrations (*C*_*o*_) and measuring the equilibrium concentrations (*C*_*e*,exp⁡_). Solving the mass balance, corresponding equilibrium loadings (*q*_*e*,exp⁡_) can be simply calculated [2]. Once pairs (*C*_*e*,exp⁡_, *q*_*e*,exp⁡_) are obtained, they are plotted and subjected to the fitting procedure. Candidate theoretical models are subsequently fitted to the experimental data (parameters of the model functions *q*_*e*,exp⁡_ = *f*(*C*_*e*,exp⁡_) are determined) and finally the best fitting model is chosen to represent the experimental system. Two different steps of the described procedure can be noticed: firstly, the method used for obtaining parameter values and, secondly, the method used for the isotherm selection.

The most commonly used empirical adsorption isotherm models are the Langmuir and Freundlich isotherms [3]. In the past decades, the equations of these two parameter functions were routinely linearized, and the parameters were directly obtained by linear regression. The preferred one among the linearized equations would have been chosen by the coefficient of determination (*R* ^2^) closer to one. Nonlinear regression, being an iterative procedure, gained popularity in the era of microcomputers. Parameter estimates in this method are obtained through minimization of the quadratic error between experimental data *q*_*e*,exp⁡_ and model outputs *q*_*e*,calc_ for all sample points. Literature survey summarized in a review paper of Foo and Hameed [4] showed that besides ordinary least squares (OLS), researchers use many other error functions, namely, hybrid fractional error function (HYBRD), Marquardt's percent standard deviation (MPSD), average relative error (ARE), and sum of absolute errors (EABS). The coefficient of determination (*R* ^2^), root mean squared error (RMSE), all of the mentioned error functions, and sometimes Akaike information criterion [5] are calculated to measure the goodness of fit and as a criterion for the selection of optimum isotherm.

However, El-Khaiary noticed that both dependent and independent variables used for construction of isotherm equations are affected by experimental errors and first used the method known as orthogonal distance regression (ODR) for the isotherm parameter estimation [6].

Having so many options open, the researcher has to decide which one to apply. The paper of El-Khaiary and Malash [7] contains the insightful analysis of the probably most common error: misuse of linearization. Studies comparing the accuracy of different error functions in predicting the isotherm parameters and the optimum isotherm are presented in the literature [8–10]. An important limitation to these earlier studies is that they have been conducted primarily on experimental data. However, there are a few drawbacks of such approach: the true, underlying isotherm function is not known and the final conclusion about which of the applied criteria has properly discovered it cannot be drawn. Also, the values of the true parameters are not known and it is not possible to decide which of the modeling approaches achieved the most accurate parameter estimates. Yet, another problem is that, even proving the validity of some method in just one particular case, one cannot easily generalize the conclusion and suggest the use of the method without sound background theory.

Valuable information can be obtained when laboratory experiments are simulated through extensive Monte Carlo calculations. This technique allows for both complete specification and absolute control of all relevant parameters, a condition that real experiments never approximate well. An advantage of Monte Carlo simulations is that they can be repeated thousands of times in a reasonable time and at very low cost.

This study was performed with the aim to answer the question which modeling approach should be applied in particular case. A few aspects of the problem were addressed. Do the isotherm equation type and number of parameters make the difference? How do the properties of the analytical method for the initial and equilibrium concentrations determination affect the parameter estimation procedure? What is the preferred method if one has some information about the measurement error structure? And what is the winning method in the case when the only available information is the isotherm dataset that consists of 5–10 points, with no replication?

The Monte Carlo technique was used as a tool to test the differences between nonlinear and orthogonal distance regression methods. Tendencies within modeling approaches were revealed on a large number of generated datasets, allowing the precision and accuracy of parameter estimates to be determined by comparison with true parameter values. Five isotherm models in the presence of five noise precision models (NPMs) were analyzed by eight modeling approaches. Three levels of reality were distinguished—theoretical level at the one side, when the noise structure is exactly known, and the two experimental levels at the other side: one in the absence of data about noise structure and the second when the estimates of standard deviations could be obtained.

As a result of this investigation, a clear strategy for data reduction in the field of adsorption is presented.

## 2. Theoretical Background

### 2.1. Adsorption Isotherms

Over the years, a wide variety of equilibrium isotherm models have been formulated. In general, an adsorption isotherm is the relationship between quantity of the component retained on a solid phase  (*q*_*e*_) and the remaining sorbate concentration in the fluid phase (*C*_*e*_), mathematically expressed as  *q*_*e*_ = *f*(*C*_*e*_). The main drawback of the isotherms is that the isotherm does not provide automatically any information about the reactions involved, and mechanistic interpretations must be carefully verified [11]. Additionally, they cannot take into account the effect of ionic strength, pH of the solution, composition of the media, and temperature. Despite these limitations, isotherms are largely employed to describe sorption phenomena. Giles et al. [12] classified isotherms as ‘‘C,” ‘‘L,” ‘‘H,” and ‘‘S,” based on the 4 main shapes of isotherms commonly observed. According to this classification, ‘‘C” isotherm is a line of zero-origin, and ‘‘L” and ‘‘H” are concave curves supported by the fact that the ratio between the concentration of the compound remaining in solution and adsorbed on the solid increases when the solute concentration increases. The ‘‘H” type isotherm is only a particular case of the ‘‘L” isotherm, where the initial slope is very high. Progressive saturation of the solid is supported by these concave isotherms and two possibilities are distinguished: the curve reaches a strict asymptotic plateau (the solid has a limited sorption capacity), and the curve does not reach any plateau (the solid does not show clearly a limited sorption capacity). ‘‘S” type of adsorption isotherm is sigmoidal-shaped and thus has got a point of inflection. It is always a result of at least two opposite mechanisms. Compared to the ‘‘L” and ‘‘H” isotherms, the ‘‘S” class occurs less frequently [13], and it will not be addressed in this paper.

From a mathematical point of view, isotherm equations can be grouped into rational, power, and transcendental functions [3]. Important for the convergence properties and computational difficulty is the number of parameters. Most of the isotherms used for liquid-phase adsorption description are two or three parameter isotherms, while for the adsorption of gases hybrid isotherms with significantly higher number of parameters are also present in the literature [14]. The equations of five adsorption isotherms addressed in this paper are listed in Table 1.

They were chosen to be widely used and to represent different types of mathematical functions (Langmuir, Redlich-Peterson, and Sips isotherms are rational functions, Freundlich isotherm is a power function, and Jovanovic isotherm is a transcendental function) and different number of parameters (Langmuir, Freundlich, and Jovanovic are two-parameter isotherms, and Redlich-Peterson and Sips are three-parameter isotherms). To avoid unnecessary repetitions, detailed characteristics of the isotherms are not presented. Additional information can be found in the literature.

### 2.2. Method of Least Squares

Let independent data pairs (*x*_*i*_, *y*_*i*_), *i* = 1,…, *n*, be observed from the underlying true values (*X*_*i*_,*Y*_*i*_), and accept the assumption that only dependent variable *y*_*i*_ is affected by measurement error:

(1) $$\begin{array}{c} x_{i}=X_{i},\\ y_{i}=Y_{i}+\varepsilon_{i}, \end{array}$$

where *ε*_*i*_ is additive, zero mean, white, Gaussian noise. The noise is assumed to be homoscedastic with constant population standard deviation *σ*_*ε*_, written in short notation *ε*_*i*_ ~ *N*(0, *σ*_*ε*_). Although (1) is not absolutely satisfied in practice, and it is often the case that *x*_*i*_ have errors, these errors can be safely ignored if they are much smaller than the corresponding errors in *y*_*i*_ [6].

Assume the smooth function *Y* = *f*(*X*; ***θ***) is a true model, where ***θ*** ∈ *R* ^*p*^ is a vector of true parameters. With more data points than parameters (*n* > *p*), it is not possible to solve the model and calculate the values of the true parameters. Instead, the question is how to obtain the best compromise so that the model predictions (${\hat{y}}_{i}$) are on the whole as close as possible to the observed data values. Closeness for any single observation may be measured by the vertical distance (*ψ*_*i*_) from the data point to the fitted curve:

(2) $$\begin{array}{c} y_{i}=\;{\hat{y}}_{i}+\psi_{i}. \end{array}$$

Closeness averaged over the entire data set is often measured by the sum of the squares of the individual distances. Any point $\hat{\theta }=\left({\hat{\theta }}_{1},{\hat{\theta }}_{2},\ldots ,{\hat{\theta }}_{p}\right)$ in Θ = {***θ*** = (*θ*_1_, *θ*_2_,…, *θ*_*p*_) ∈ **R** ^*p*^, *θ*_*i*_ > 0, *i* = 1,…, *p*} which minimizes the functional,

(3) $$\begin{array}{c} \text{ErrFun}\left(\hat{\theta }\right)=\overset{n}{\underset{i=1}{\sum }}w_{i}^{2}{\left(f\left(x_{i};\hat{\theta }\right)-y_{i}\right)}^{2}, \end{array}$$

where *w*_*i*_ are data weights and equation for the fitted curve reads ${\hat{y}}_{i}=f(x_{i};\hat{\theta })$, is called the least squares estimate of the unknown parameters, if it exists [20]. Condition *θ*_*i*_ > 0, *i* = 1,…, *p*, in the definition of the set Θ is a specific feature of the modeling adsorption isotherms and meets the criterion for the isotherm to be positive, increasing, and concave on the set [0, ∞), namely, to be ‘‘L” or ‘‘H” type isotherm.

#### 2.2.1. Weighting Schemes in the Method of Least Squares

In the method of OLS the observations are assumed to be homoscedastic and all of the points are assigned equal weights *w*_*i*_ = 1, *i* = 1,…, *n*. In the absence of more complete information, it is commonly accepted that uniform weighting is satisfactory, and OLS are widely used in model fitting [21]. If the assumption of constant standard deviations of measurement errors is relaxed, the heteroscedasticity is characterized by an *n* element vector **σ**_**ε**_ = (*σ*_*ε*1_, *σ*_*ε*2_,…, *σ*_*εn*_), where each *σ*_*εi*_ is population standard deviation of the noise at *y*_*i*_ : *ε*_*i*_ ~ *N*(0, *σ*_*εi*_). Weights calculated by

(4) $$\begin{array}{c} w_{i}=\frac{1}{\sigma_{\varepsilon i}} \end{array}$$

are introduced into (3) in order to account for inconstant variance and the method is referred to as weighted least squares (WLS) or sometimes “chi-square fitting” [22]. The assumption that the weights are known exactly is not valid in real applications so estimated weights must be used instead [23].

Ideally, observation weights should be estimated according to individual estimates of measurement error such that *w*_*i*_ = 1/*sdy*_*i*_, where *sdy*_*i*_ is the standard deviation of *i*th measurement. These are called instrumental weights.

When individual error estimates are unavailable, other empirical weights may provide a simple approximation of standard deviation. For the peculiar case of heteroscedasticity important in many analytical methods, relative standard deviations are reasonably constant over a considerable dynamic range. Thus, *σ*_*εi*_ is proportional to *y*_*i*_ and the weights can be estimated as *w*_*i*_ = 1/*y*_*i*_.

However, the error structure in real data usually lies somewhere on a continuous between a constant absolute error (homoscedastic) at one extreme and a constant percentage error at the other. Between these two there is an error for which the standard deviation is proportional to the square root of the expected value: *w*_*i*_ = 1/*y*_*i*_^0.5^. This type of weights is called Poisson weights or hybrid weights and they should be applied when the shot noise is present. Shot noise is dominant when a finite number of particles that carry energy (ions, electrons, and photons) are counted at the detector part of the instrument. The characteristic expressions for each weighting type are presented in Table 2.

ISOFIT, a software package for fitting sorption isotherms to experimental data by weighted least squares, supports three alternatives: uniform weighting, sorbed relative where weights are inversely proportional to sorbed concentrations, and solute relative where weights are inversely proportional to measured solute concentrations [24].

### 2.3. Orthogonal Distance Regression Methods

In a more general situation, considerable errors can occur in both variables. It is stated that if the errors in *x*_*i*_ are greater than one-tenth of the errors in *y*_*i*_, then the overall error is significantly increased. Moreover, the regression parameters and their confidence intervals are then biased using (ordinary) weighted least squares [25].

Let the considerable error be also present in the measurements of the independent variable

(5) $$\begin{array}{c} x_{i}=X_{i}+\delta_{i}, \end{array}$$

where *δi* ~ *N*(0, *σ*_*δ*_*i*__).

Again, the model will not fit the observed data points (*x*_*i*_, *y*_*i*_), *i* = 1,…, *n*, exactly, so the corresponding set of points $\left({\hat{x}}_{i},{\hat{y}}_{i}\right)$, *i* = 1,…, *n*, that do fit the model exactly and that are at the same time the closest to experimental data points is to be considered. For each data point, the value of independent variable ${\hat{x}}_{i}$ is expressed introducing an error term *φ*_*i*_:

(6) $$\begin{array}{c} {\hat{x}}_{i}=x_{i}+\varphi_{i}. \end{array}$$

The values ${\hat{y}}_{i}$ are predicted by the model function

(7) $$\begin{array}{c} {\hat{y}}_{i}=f\left(x_{i}+\varphi_{i};\hat{\theta }\right). \end{array}$$

A reasonable way to estimate the unknown parameters in this case is to minimize the weighted sum of squares of all errors by minimizing the functional:

(8) $$\begin{array}{c} \text{ErrFun}\left(\hat{\theta },\hat{\varphi }\right)=\overset{n}{\underset{i=1}{\sum }}w_{i}^{2}\left({\psi_{i}}^{2}+d_{i}^{2}\varphi_{i}^{2}\right), \end{array}$$

on the set Θ × Φ, where *w*_*i*_ and *d*_*i*_ are data weights in the *y* and *x* directions, respectively, and

(9) $$\begin{array}{c} \Phi ∶=\left\{\varphi =\left(\varphi_{1},\varphi_{2},\ldots ,\varphi_{n}\right)\in R^{n},x_{i}+\varphi_{i}\geq 0,i=1,\ldots ,n\right\}. \end{array}$$

Commonly, (8) is expressed in its expanded form, where the difference between calculated and experimentally observed value of *y* is emphasized:

(10) $$\begin{array}{c} \text{ErrFun}\left(\hat{\theta },\hat{\varphi }\right)=\overset{n}{\underset{i=\;1}{\sum }}w_{i}^{2}\left[{\left(f\left(x_{i}+\varphi_{i};\hat{\theta }\right)-y_{i}\right)}^{2}+d_{i}^{2}\varphi_{i}^{2}\right]. \end{array}$$

This approach is known as errors in variables or orthogonal distance regression or total least squares. Condition *x*_*i*_ + *φ*_*i*_ ≥ 0, *i* = 1,…, *n*, in the defining relation (9) is set in order to meet the natural condition that concentration is a nonnegative value.

#### 2.3.1. Weighting Schemes in the Method of Orthogonal Distance Regression

In orthogonal distance regression analysis of sorption data, units of the variables on the axes are not the same. It is necessary to introduce weights as constants selected to scale each type of variable *y*_*i*_ or *x*_*i*_. This is done in order to put the model errors (*ψ*_*i*_ and *φ*_*i*_) on a comparable basis, so it will be meaningful to add them all together into the sum of error function in (8). Typically, weights are chosen as estimates of the population standard deviation of the experimental measurements of each variable type: *w*_*i*_ = 1/*sdy*_*i*_ and *w*_*i*_ *d*_*i*_ = 1/*sdx*_*i*_. Another way is to assign weights to be proportional to the inverse of experimental values: *w*_*i*_ = 1/*y*_*i*_ and *w*_*i*_ *d*_*i*_ = 1/*x*_*i*_. The effect of such weights is that at the same time heteroscedasticity is accounted for and scaled model errors in *y* and *x* direction are dimensionless.

**Figure 1:**
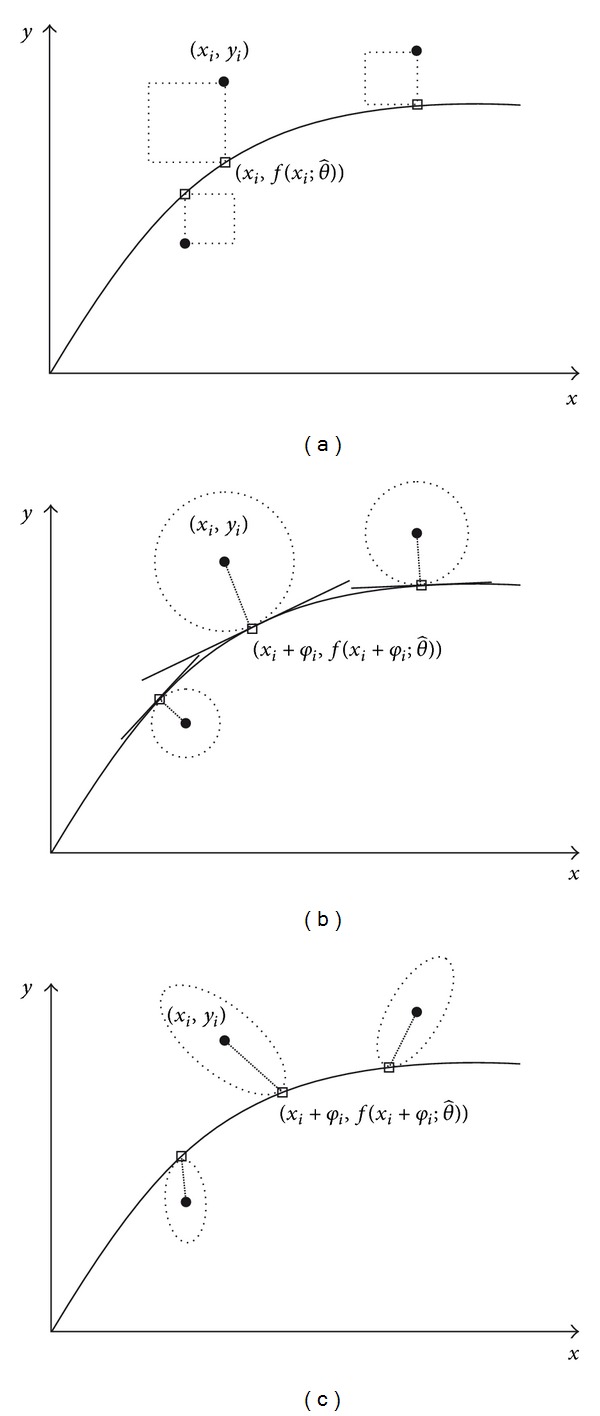
Geometric illustration of differences among different regression methods: (a) OLS without weighting, (b) orthogonal distance regression without weighting, and (c) orthogonal distance regression with weighting.

In Figure 1 differences between different OLS and orthogonal distance regression methods with and without weighting are presented.

Geometrically, if the data pairs (*x*_*i*_, *y*_*i*_) and the curve  $\hat{y}=f(\hat{x};\hat{\theta })$ are presented in the Cartesian coordinates in ordinary least squares, minimization of the error function corresponds to minimization of the shortest distances from data points to a line in a direction that is parallel to the vertical axis.  Figure 1(a) is a standard geometric illustration of the least squares method. Instead of the vertical offsets, the shortest distances from points to the line are considered in the method of orthogonal distance regression. If the data are homoscedastic, and the units of *x* and *y* are the same, all the weights *w*_*i*_ and *d*_*i*_ are equal to one. Equation (8) is then simplified to a formula that possesses a meaning of the sum of the areas of the circles shown in Figure 1(b):

(11) $$\begin{array}{c} \text{ErrFun}\left(\hat{\theta },\hat{\varphi }\right)=\overset{n}{\underset{i=1}{\sum }}\left[{\left(y_{i}-{\hat{y}}_{i}\right)}^{2}+{\left(x_{i}-{\hat{x}}_{i}\right)}^{2}\right]. \end{array}$$

In this case, the radii of these circles are equal to distances between the points (*x*_*i*_, *y*_*i*_) and the fitting line. Put in other words, the fitting line is a tangent line to all circles.

Geometrical representation of a case when *x* and *y* are variables that do not have the same units or the data is heteroscedastic is presented in Figure 1(c). Weights are introduced and half axes of the ellipses in Figure 1(c) correspond to the combined measure of the distance expressed in (8). While the global minimum of this error function is unique, this kind of straightforward geometrical representation is no longer meaningful.

Orthogonal distance regression methods have been used in the fields of science such as economy [26], automatic control [27], and pharmacology [28], and a significant work has been done for the development of stable and efficient algorithm for ODR estimation of parameters.

## 3. Materials and Methods

### 3.1. Numerical Experiments

Numerical experiments were designed to be as close as possible representation of a typical experimental setup in adsorption studies. It was adopted that batch experiments are performed in laboratory bakers containing mass of sorbent *m* and volume *V* of sorbate solution. Initial concentrations of sorbate solutions (*C*_*oi*,true_) are chosen to be 0.5, 1.0, 5.0, 10.0, 50.0, and 100.0. All units are ignored since they are irrelevant. Further on, it was assumed that the theoretical adsorption isotherm expressed in terms of its true parameters is exactly matching the adsorption process. Values of the true parameters were arbitrarily set to get the operative expression *q*_*e*,true_ = *f*(*C*_*e*,true_), where *C*_*e*,true_ is errorless equilibrium sorbate concentration and *q*_*e*,true_ is errorless equilibrium sorbate loading. At the same time, mass balance expressed in (12) is satisfied:

(12) $$\begin{array}{c} q_{ei,\text{true}}=\frac{\left(C_{oi,\text{true}}-C_{ei,\text{true}}\right)}{m}V.\; \end{array}$$

The true equilibrium concentration is then calculated solving the equation:

(13) $$\begin{array}{c} f\left(C_{ei,\text{true}};\theta \right)-\frac{\left(C_{oi,\text{true}}-C_{ei,\text{true}}\right)}{m}V=0. \end{array}$$

It is assumed that simple univariate chemical measurement system with additive, zero mean, white Gaussian measurement noise is used as an analytical tool to determine *C*_*oi*,true_ and *C*_*ei*,true_. Thus random noise (*δ*_*i*^*o*^_ ~ *N*(0, *σ*_*δi*_^*o*^) and *δ*_*i*^*e*^_ ~ *N*(0, *σδ*_*i*^*e*^_), resp.) was added to these values to obtain simulated experimental concentrations:

(14) $$\begin{array}{c} C_{oi,\exp}=C_{oi,\text{true}}+\delta_{i}^{\text{o}},\\ C_{ei,\exp}=C_{ei,\text{true}}+\delta_{i}^{e}. \end{array}$$

The rest of the procedure was identical as if the experiments were performed in laboratory. The equilibrium sorbent loading was calculated from (12), and the collected data was subjected to fitting routines.

**Figure 2:**
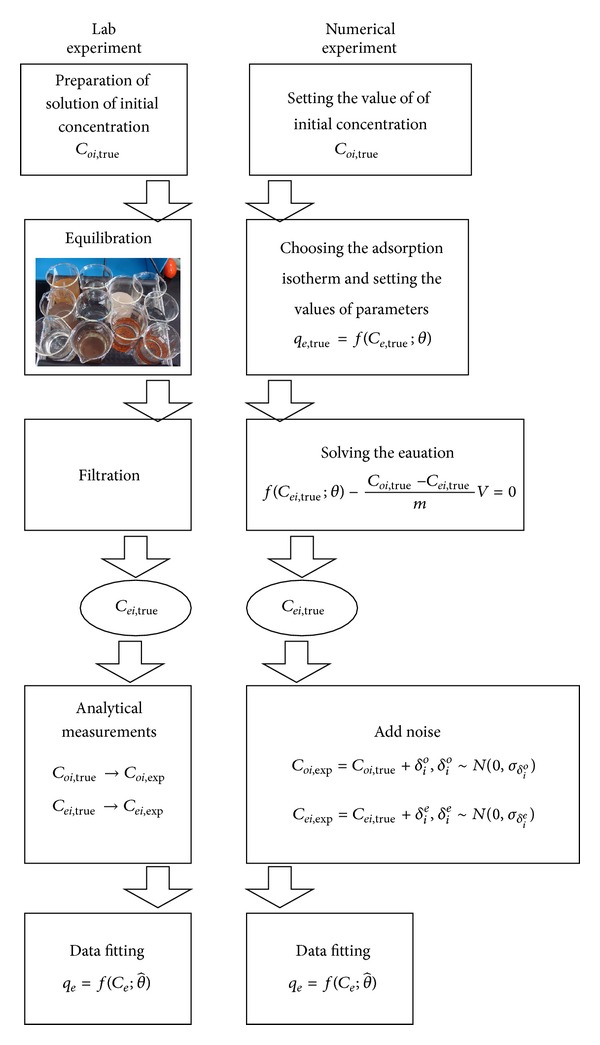
Flow chart illustrating the steps in adsorption equilibrium experiment and a matching numerical experiment.

Flow chart of laboratory experiment and a matching numerical experiment is presented in Figure 2.

### 3.2. Noise Precision Models

Since a wide variety of substances (toxic metals, organic pollutants, etc.) are in focus of adsorption research community, also a wide variety of analytical techniques are used for initial and equilibrium concentrations determination. Accordingly, the measurement errors they introduce differ in type and magnitude. There are different mathematical models, named noise precision models (NPMs), that have been proposed to estimate the change of analytical precision as a function of the analyte concentration. List of such models for specific analytical methods, together with explanations of error sources, can be found in literature [23]. In this paper, the NPMs were chosen to be the simplest physically plausible [29]. Firstly, the six different magnitudes of homoscedastic noise were used (H1–H6). Noise population standard deviations were from very low (0.01, 0.02) and medium (0.05, 0.1) to high (0.2, 0.4). The noise of the data was therefore between 0.01% and 0.4% of the data range. Secondly, the five types of heteroscedastic noise, linear (Lin), quadratic (Quad), hockey stick (HS), constant relative standard deviation of 5% (RSD5%), and constant relative standard deviation of 10% (RSD10%), were involved. Expressions of the NPMs used in the study and their relevant parameters are listed in Table 3.

### 3.3. Methods for Fitting Adsorption Isotherms

Although there are some experiments where it is reasonable to assume that one variable (*x*) is largely free from errors, such an assumption is manifestly not true in cases of adsorption isotherms. The value on the *x*-axis is an equilibrium concentration, *C*_*e*,exp⁡_, which is determined by chemical analysis and thus inevitably affected by the measurement error. Equilibrium loading, *q*_*e*,exp⁡_, (which is on the *y*-axis) is calculated from the equilibrium concentration, and as a result an error in this concentration appears in both coordinates. Particular attention must be given to equations in which one variable is involved on both sides, since the independence of errors is not fulfilled [30]. The use of orthogonal distance regression modeling procedure would be statistically correct in this case.

Since this study is based on simulated data, population standard deviations of measurement errors are known and (10) can be transformed into theoretical orthogonal distance regression criterion (TODR) introducing the relations

(15) $$\begin{array}{c} w_{i}=\frac{1}{\sigma_{\varepsilon i}},\\ w_{i}d_{i}=\frac{1}{\sigma_{\delta i}}, \end{array}$$

for the weights on the *y*- and *x*-axes, respectively. It can be noticed that the population standard deviation of the error on the *x*-axis is actually the population standard deviation of the equilibrium concentration:

(16) $$\begin{array}{c} \sigma_{\delta i}=\sigma_{\delta i}^{e}. \end{array}$$

For the *y* axis, *σ*_*εi*_ is calculated based on (12). According to the error propagation law

(17) $$\begin{array}{c} \sigma_{\varepsilon i}^{2}=k^{2}\left[{\left(\sigma_{\delta i}^{e}\right)}^{2}+{\left(\sigma_{\delta i}^{o}\right)}^{2}\right], \end{array}$$

where *k* = *V*/*m*, it is assumed that only the error of the *C*_*o*,exp⁡_ and *C*_*e*,exp⁡_ determination is significant, while mass and volume can be accurately and precisely measured.

Final formulation of the TODR error function which is minimized in case of theoretical fitting of adsorption data is presented as

(18) $$\begin{array}{c} \text{TODR}=\overset{n}{\underset{i=1}{\sum }}\left[{\left(\frac{q_{ei,\exp}-q_{ei,\text{cal}}}{\sigma_{\varepsilon i}}\right)}^{2}+{\left(\frac{C_{ei,\exp}-C_{ei,\text{cal}}}{\sigma_{\delta i}^{e}}\right)}^{2}\right]. \end{array}$$

Although TODR cannot be used outside the theoretical domain, it was included in this study to serve as a golden standard. It is expected to represent the best possible results that can be achieved with the certain observations in possession.

A typical isotherm data set in the experimental domain consists of 5–10 points. Very often, researches perform their experiments in triplicate [31–33], but in cases when the reagents are expensive or toxic there are no replications. By these two different methods, different levels of data quality are obtained. The objective of this paper was to take both of these cases into consideration.

Data obtained in only one numerical experiment (matching the case when the laboratory experiments are performed with no replication) were fitted by the use of four error functions: OLS, ODR, MPSD, and HYBRD. Their expressions are presented in Table 4. Least squares fitting of the linearized data (LIN) was additionally applied only on the Langmuir and Freundlich equations.

Formulae of the error functions in Table 4 are a bit different from the ones reviewed in [4]. The key reason is that the number of data points is constant all the time, and there will be no isotherm ranking based on this error functions, so the constants n-p were removed for the sake of simplicity. Parameter estimates obtained with these slightly modified error functions are the same because the multiplication of error function with a constant nonzero value does not affect the position of a global minimum.

OLS, MPSD, and HYBRD are basically least squares methods with different types of weights included. OLS is the approach with all weights equal to one. In case of MPSD, assumption of constant percentage error is accepted and weighting by the equilibrium loading is applied. For the HYBRD error functions weights are of the Poisson type. ODR abbreviation in this context is used for the orthogonal distance regression analog of the MPSD. Assumption of constant percentage error is accepted for both of the axes, and the weights are 1/*q*_*e*,exp⁡_ and 1/*C*_*e*,exp⁡_ for the *y*- and *x*-axes, respectively.

The second group of calculations matched the case when laboratory experiments are performed in triplicate. Means of equilibrium sorbent loading (${\overset{-}{q}}_{ei,\exp}$) and equilibrium sorbate concentration (${\overset{-}{C}}_{ei,\exp}$) from three subsequent numerical experiments were passed to the following error functions: experimental weighted orthogonal distance regression (E3WODR), triplicate orthogonal distance regression (E3ODR), and weighted least squares (WLS). Definitions of error functions for replicated measurements are listed in Table 5.

It is important to say that E3WODR is the experimental realization of TODR. Estimates of standard deviation of the variables on the *y*- and *x*-axes are calculated as described in Section 2.3.1 incorporating the following equations:

(19) $$\begin{array}{c} sdy_{i}=\sqrt{\overset{3}{\underset{j=1}{\sum }}\frac{{\left(q_{ei,\exp}-{\overset{-}{q}}_{ei,\exp}\right)}_{j}^{2}}{2},} \end{array}$$

(20) $$\begin{array}{c} sdx_{i}=\sqrt{\overset{3}{\underset{j=1}{\sum }}\frac{{\left(C_{ei,\exp}-{\overset{-}{C}}_{ei,\exp}\right)}_{j}^{2}}{2}.} \end{array}$$

Weighting in the E3ODR method is based on the mean values of equilibrium sorbent loading and equilibrium sorbate concentration:

(21) $$\begin{array}{c} w_{i}=\frac{1}{{\overset{-}{q}}_{ei,\exp}},\\ w_{i}d_{i}=\frac{1}{{\overset{-}{C}}_{ei,\exp}}. \end{array}$$

For the WLS method, instrumental weights are calculated based on (19).

### 3.4. Numerical Calculations

The present work was carried out using Windows-based PC with hardware configuration containing the dual processor AMD Athlon M320 (2.1 GHz each) and with 3 GB RAM. All calculations were performed using Matlab R2007b. Perturbations were generated using Mersenne Twister random number generator. For the purpose of fitting, built-in Matlab function fminsearch was used for OLS, MPSD, HYBRD, and WLS [34]. It is based on the Nelder-Mead simplex direct search algorithm. Orthogonal distance regression calculations used the dsearchn function as a tool to find the set of $\left({\hat{x}}_{i},{\hat{y}}_{i}\right)$ values.

It is important to note that one complete numerical experiment and all associated computations were performed for each simulation step, for 2000 steps per combination (one type of isotherm and one type of NPM). The reason simulations were chosen to have 2000 steps was the compromise between the aim to have resulting histograms of quality high enough to facilitate quantitative comparison with theory and to prevent the process from lasting unacceptably long. Few of the simulations took longer than 12 hours, with the most averaging around 8 hours in length. It could be noticed that simulations with higher values of noise standard deviation in general lasted longer. The explanation is the rise in the number of function evaluations and the number of iterations before the convergence is achieved.

## 4. Results and Discussion

### 4.1. Postprocessing of the Results

This study included 55 numerical simulations on the whole (each of the five isotherms presented in Table 1 was paired with the 11 noise precision models presented in Table 3) and they were individually subjected to the same postprocessing routine. The raw results of each numerical simulation were 2000 (two or three element) vectors of estimated parameters per modeling approach. Firstly, the cases when the particular fitting algorithm did not converge were counted, and corresponding parameter estimates were removed from further consideration. The second step in postprocessing procedure was to calculate percentage errors for all the parameter estimates:

(22) $$\begin{array}{c} e_{l,j}=\frac{{\hat{\theta }}_{l,j}-\theta_{l}}{\theta_{l}}100\%, \end{array}$$

where *l* is the parameter ordinal number in the isotherm equation (*l* ∈ {1, 2} for the Langmuir, Freundlich, and Jovanovic isotherms and *l* ∈ {1, 2, 3} for Redlich-Peterson and Sips equations) and *j* is the number of numerical experiments. The third step was to identify and remove the outlier values. An *e*_*l*,*j*_ value was considered as an outlier if it is greater than 75th percentile plus 1.5 times the interquartile range or if it is less than 25th percentile minus 1.5 times the interquartile range. The outliers were removed to once again match the simulated and real cases. It is common practice to discard the fitting results if they do not correspond to common sense. Sets of percentage error on parameters obtained in the described way were used for statistical evaluation.

### 4.2. Statistical Evaluation

**Figure 3:**
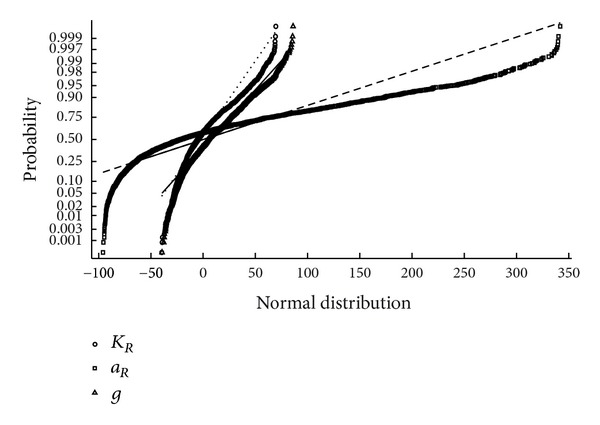
Normal probability plot for the parameters of Redlich-Peterson isotherm and OLS processing.

The normal probability plots were used to graphically assess whether the obtained parameter estimates could come from a normal distribution. Inspection of such plots showed that in general they are not linear. Distributions other than normal introduce curvature, so it was concluded that nonnormal distribution is involved. One representative example is presented in Figure 3.

Thus, median of percentage error (mE) was used as a measure of accuracy of the method based on a particular error function, and mean absolute relative error (MARE),

(23) $$\begin{array}{c} \text{MARE}=\frac{1}{kf}\overset{kf}{\underset{j=1}{\sum }}\left|e_{l,j}\right|, \end{array}$$

where *kf* is the number of converged fits for the particular method in a simulation, was used as a measure of precision. The individual performance of each modeling approach was evaluated for each isotherm and for each type of noise.

Comparison of the methods was done separately for the two following groups of data: observations with no replications and data from triplicate experiments.

**Figure 4:**
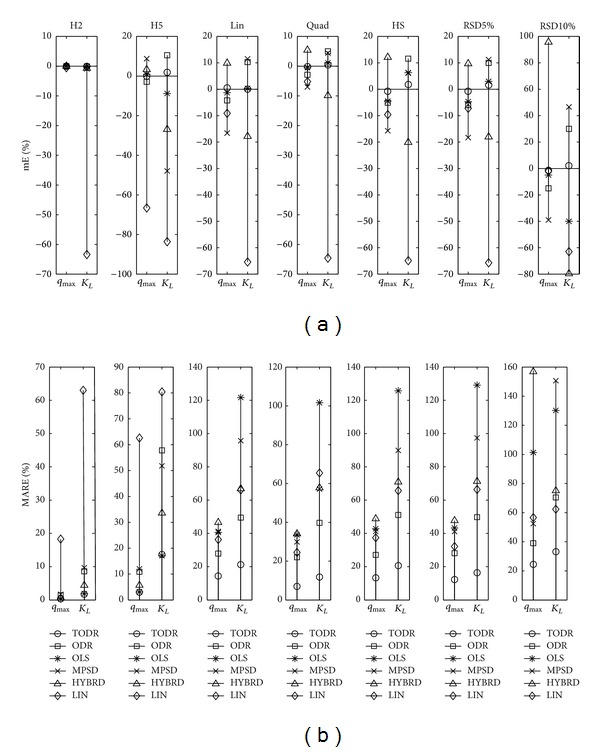
Properties of different modeling approaches for determination of parameters in Langmuir isotherm (experiments performed once): (a) mE and (b) MARE.

**Figure 5:**
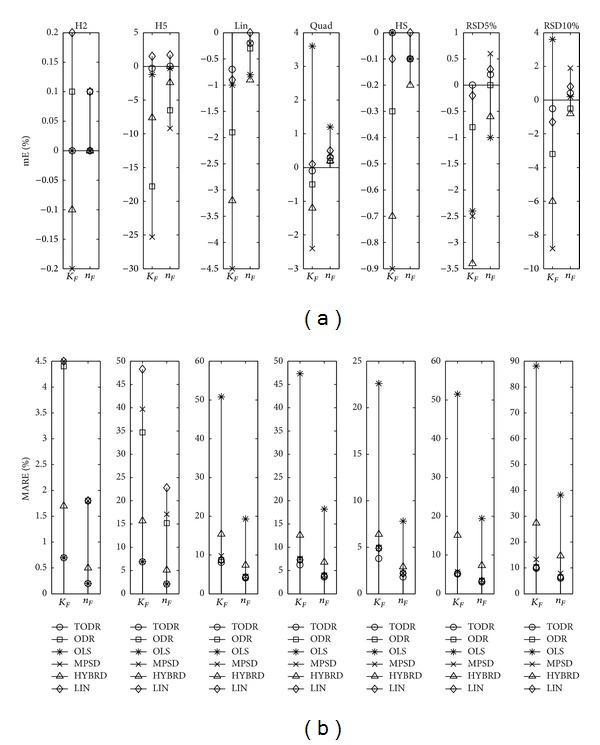
Properties of different modeling approaches for determination of parameters in Freundlich isotherm (experiments performed once): (a) mE and (b) MARE.

**Figure 6:**
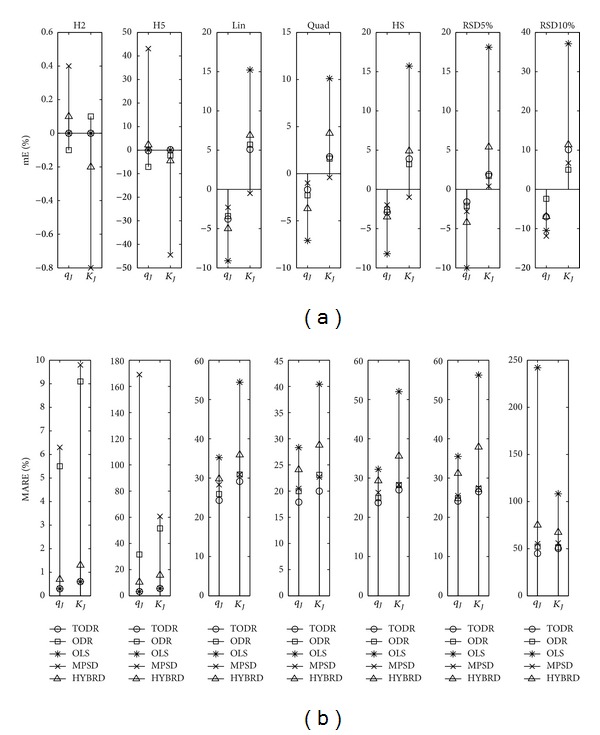
Properties of different modeling approaches for determination of parameters in Jovanovic isotherm (experiments performed once): (a) mE and (b) MARE.

**Figure 7:**
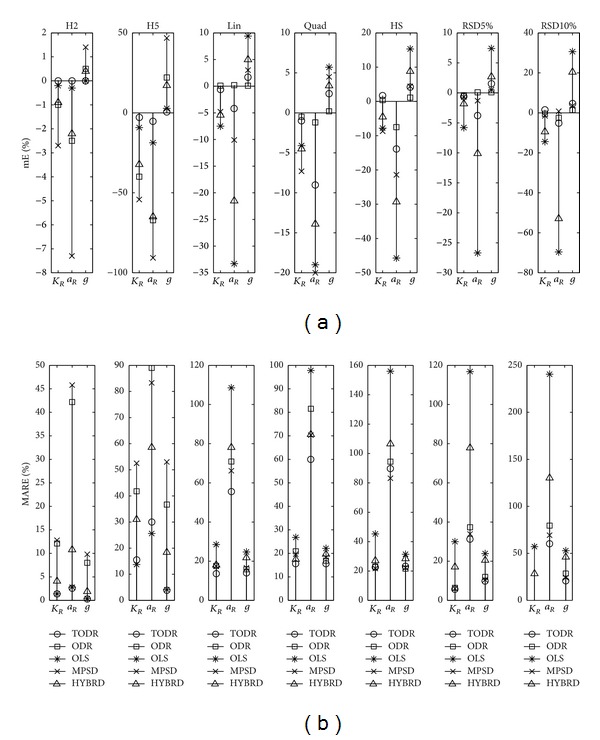
Properties of different modeling approaches for determination of parameters in Redlich-Peterson isotherm (experiments performed once): (a) mE and (b) MARE.

**Figure 8:**
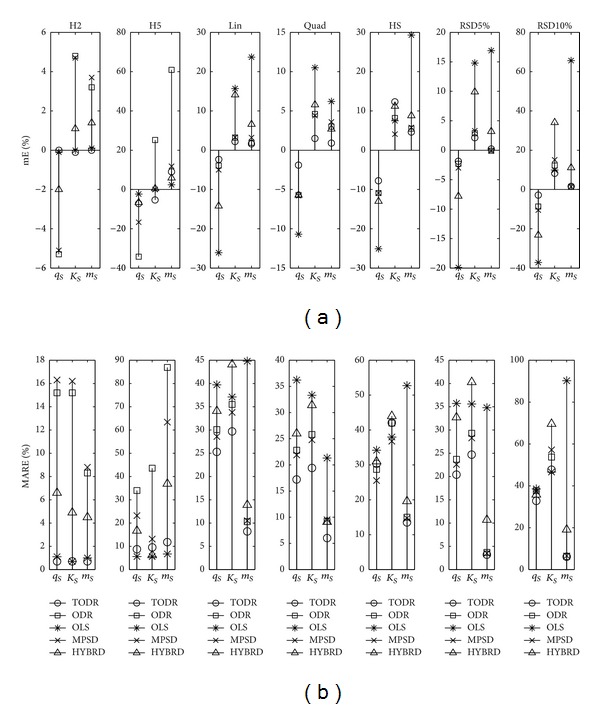
Properties of different modeling approaches for determination of parameters in Sips isotherm (experiments performed once): (a) mE and (b) MARE.

Properties of different modeling approaches in case when the experiments are performed once are presented in Figures 4, 5, 6, 7, and 8 for the Langmuir, Freundlich, Jovanovic, Redlich-Peterson, and Sips adsorption isotherms, respectively.

Properties of different modeling approaches in case when the experiments are performed three times are presented in  Figures A.1–A.5 as in Supplementary Material available online at http://dx.doi.org/10.1155/2014/930879, for the Langmuir, Freundlich, Jovanovic, Redlich-Peterson, and Sips adsorption isotherms, respectively.

Due to a huge quantity of results obtained in this study, some rules had to be put on what is going to be presented in figures. For every type of isotherm, the figures are organized to have two sections: one where mE values are presented (figures labeled (a)) and the other where MARE values are presented (figures labeled (b)). Each section of the plot contains 7 subplots. In one subplot, the results of the applied methods for one NPM are summarized. Trends were noticed and discussed based on the six levels of homoscedastic noise, but in order to make figures compact just two out of six NPMs (H2 as an example of low noise, and H5 as an example of high noise) were presented as the first two subplots. The next five (3–7) subplots were reserved for heteroscedastic NPMs. An additional remark is valid for all the figures in the following paragraph: it was not possible to use the same scale in all subplots, due to large differences in the magnitudes of the outcomes from subplot to subplot. Nevertheless, it does not introduce any problem because the comparison of methods is done in frames of a subplot, and cross comparisons between different NPMs (and subplots) are not of substantial importance.

#### 4.2.1. Experiments with No Replications and Homoscedastic Noise

As expected, for the very low level of homoscedastic noise (H1 and H2 noise precision models), all of the examined methods performed well. With the increasing of noise standard deviation, the accuracy and precision of the methods became worse, and differences between methods started to appear.

Generalizing the results of all the five isotherms, the following statements can be placed. Regardless of the mathematical type of isotherm equation (rational, power, or exponential) and the number of parameters (two or three), the OLS method had the best properties. In the group of methods applicable in practice, it achieved mE values closest to zero and the lowest values of MARE, almost identical to ones determined by the theoretical method TODR. mE of the other tested methods showed higher discrepancy from zero, and MARE values were higher. ODR and MPSD methods had a very bad performance, while the results of the HYBRD method were somewhere in between.

Closeness of the results of OLS and TODR methods showed that in case of homoscedastic noise, the presence of measurement error on both axes is not of great importance, as it could be expected. What is more, the weighting by 1/*q*_*e*,exp⁡_ and 1/*C*_*e*,exp⁡_ for the *y*- and *x*-axes, respectively, in the ODR method is actually wrong since the population standard deviations are constant at all concentrations. That is the reason why the ODR method is biased and of low precision. In the recent study, the different modeling approaches were tested on a Langmuir isotherm with perturbations of data with the fixed error *N*(0, 0.05) and with ±5% error proportional to concentration [35]. Surprisingly, authors found that the ODR gives the most accurate estimates (lowest mean, standard deviation, and interquartile range) of the isotherm parameters among different methods when the experimental data have a fixed error.

For the two parameter isotherms (Langmuir and Freundlich), linearized models were tested due to their popularity. Modeling of linearized Langmuir equation was the only exception from the rule that the greater the population standard deviation of the noise, the greater the discrepancies of parameter estimates from their true values. Regardless of the level of noise, the LIN method presented equally bad results. The mE was about −65% and MARE in the range 60–85% for both of the parameters. For the Freundlich isotherm, LIN model was more accurate than HYBRD, MPSD, and ODR and had about the same variability as MPSD, still resigning in the group of modeling approaches whose usage is not advised in the case of homoscedastic data.

#### 4.2.2. Experiments with No Replications and Heteroscedastic Noise

Looking at the subplots 3–7 of Figures 5–8 where the results of modeling Freundlich, Jovanovic, Redlich-Peterson, and Sips isotherm are presented, the pattern of the OLS method performance can be easily recognized. For all of the isotherms and all of the tested NPMs, this method was the poorest of all the methods, in both the aspects of its accuracy and precision. This is the expected result because the basic assumptions when the OLS method is valid are not met. It is interesting to note that for the Langmuir isotherm (Figure 4, subplots 3–7), OLS method was the most accurate method, but the precision was still very, very poor (always more than 35% for the *q*_max⁡_ and more than 100% for the *K*_*L*_). The same as for the other isotherm types, the use of OLS for the Langmuir isotherm is not advised in the presence of any type of heteroscedastic noise.

The ODR method was generally as accurate as TODR. The greatest deviation of mE from zero was −3.2% for the RSD10% noise type and *K*_*F*_ parameter of Freundlich isotherm, 5.7% for the Lin noise type and *K*_*J*_ parameter of Jovanovic isotherm, −7.5% for the HS noise type and *a*_*R*_ parameter of Redlich-Peterson isotherm, and −10.9% for the HS noise type and *q*_*S*_ parameter of Sips isotherm. Only for the Langmuir isotherm, difference between mE of the ODR and TODR was more pronounced, reaching the absolute maximum value of 30.0% for *K*_*L*_. The MARE of ODR was higher than the MARE of TODR but the lowest among the other tested methods. Typical values of MARE of Langmuir isotherm were in the range 27%–39% for *q*_max⁡_ and 39.7%–70.4% for *K*_*L*_. For the Freundlich isotherm, MARE was below 10% for both of the parameters and all types of NPMs. The precision in case of Redlich-Peterson isotherm was roughly about 20% for *K*_*R*_ and *g* and two or three times higher for the *a*_*R*_. For the Sips isotherm, the maximum MARE was 53.7% for *a*_*R*_ in case of RSD10%, but most of the time it was roughly about 30%. Direct comparison of ODR and MPSD qualified MPSD as the second best choice method. Its accuracy and variability were at the same level or closely followed the ODR. The HYBRD method was neither accurate nor precise, again performing between the worst (OLS) and the best (ODR) methods. The same can be stated for the LIN method of the Langmuir and Freundlich isotherms.

#### 4.2.3. Experiments Performed in Triplicate

In case when the equilibrium adsorption experiments are performed once and the estimates of standard deviation of the measurement error are not available weighting is restricted to be fixed or to be some function of measured variable. When the experiments are done in triplicate, this restriction is released since the estimates of standard deviations could be obtained. The adsorption literature, surprisingly, rarely takes into account these important statistical details related to the processing of data in regression analysis with replicate measurements. Commonly, but not properly, data from triplicate measurements are just averaged, and their mean values are further on processed like OLS. Weighted regression is a way of preserving the information and thus should be preferred.

In  Figures A.1–A.5 (in Supplementary Material), the results of modeling Langmuir, Freundlich, Jovanovic, Redlich-Peterson, and Sips isotherm are presented. At first glance, it can be noticed that the accuracy and precision of the methods are better than in case of one experiment per point.

The E3WODR method had the best properties, WLS performed slightly worse, and E3ODR was ranked the third. The exception was only the Freundlich equation, where MARE values tended to lower for E3ODR method in case of heteroscedastic noise. However, difference between E3WODR and E3ODR was less than 1%, and thus this particular behavior is not of great importance.

## 5. Conclusions and Recommendations

The accuracy of model parameters will depend on whether the appropriate conceptual model was chosen, whether the experimental conditions were representative of environmental conditions, and whether an appropriate parameter estimation method was used.

Recently our group faced the problem of modeling the adsorption isotherms [36, 37] and the intention of this study was to single out the method of parameter estimation which is suitable for adsorption isotherms. A detailed investigation has been carried out to determine whether the mathematical type of isotherm function and the type of measurement noise (homoscedastic or heteroscedastic) are key factors that lead to modeling approach choice. Commonly used methods: OLS and least squares of linearized equations, methods that are far less present in literature: HYBRD and MPSD, and a method that is hardly ever used in adsorption field: orthogonal distance regression, were compared. Evaluations of these methods were conducted on a large number of data sets, allowing precision and accuracy of parameter estimates to be determined by comparison with true parameter values.

It was demonstrated that trends that could be noticed do not show dependence on isotherm type. Only the magnitude of percent errors in parameter estimates classifies some of the equation types and their particular parameters as difficult to fit (*a*_*R*_ in Redlich-Peterson isotherm). It was shown that the impact of measurement error noise type is significant. Neglecting the information about noise structure can lead to biased and/or imprecise parameter estimates and the researcher should obtain the information about the measurement error type of the analytical method used for concentration determination. (Testing the analytical system for heteroscedasticity should be a part of validation protocol necessary for the limit of detection calculation [38, 39].) As expected, OLS method performed superior in case of homoscedastic noise, no matter whether the noise is high or low. The results for heteroscedastic noise types revealed the potential of using ODR method, with weighting proportional to 1/*q*_*e*,exp⁡_ and 1/*C*_*e*,exp⁡_ for the *y*- and *x*-axes, respectively. In this situation, use of this method resulted in smaller bias and better precision. Although frequently being used in present adsorption literature for fitting adsorption isotherms, OLS method is an unfavorable method for the heteroscedastic data, performing much worse than other nonlinear methods.

The accuracy and variability of orthogonal distance regression-based methods (ODR for experiments performed once and E3ODR and E3WODR for experiments preformed in triplicate) are closely followed by the analog methods that do not take into account the influence of measurement error on both axes: MPSD and WLS.

Linearization of isotherm equations was once again discarded in this study. Since the survey of the literature published in last decade showed that in over 95% of the liquid phase adsorption systems the linearization is the preferred method [4], a lot of attention should be put on education and spreading the right principles among researches.

Further research that is currently in progress in our group will hopefully resolve the issue of adequate model selection in adsorption studies.

## Supplementary Material

Graphical representation of the results obtained in case when adsorption data is collected in repeated experiments is presented in Supplementary material. Properties of applied methods are compared in terms of median of percentage error and mean absolute relative error for the isotherm parameter determination.

## Figures and Tables

**Table 1:** Adsorption isotherm models.

No.	Type	Type of function	Nonlinear form	Linear form	True parameters			Reference
					**θ**_ 1_	**θ**_ 2_	**θ**_ 3_	
1	Langmuir	Rational	$q_{e}=\frac{q_{\max}K_{L}C_{e}}{1+K_{L}C_{e}}$	$\frac{1}{q_{e}}=\frac{1}{q_{\max}K_{L}}\frac{1}{C_{e}}+\frac{1}{q_{\max}}$	*q*_max⁡_ = 0.6	*K*_*L*_ = 0.4	/	[15]
2	Freundlich	Power	*q*_e_ = *K*_F_ *C*_e_^1/*n*_*F*_^	$\ln q_{e}=\ln K_{F}+\frac{1}{n_{F}}\ln C_{e}^{}$	*K*_*F*_ = 0.1	*n* = 1.2	/	[16]
3	Jovanovic	exp*	*q*_e_ = *q*_J_(1 − *e* ^−*K*_J_*C*_e_^)	/	*q*_*J*_ = 5.1	*K*_*J*_ = 0.02	/	[17]
4	Redlich-Peterson	Rational	$q_{e}=\frac{K_{R}C_{e}}{1+a_{R}C_{e}^{g}}$	/	*K*_*R*_ = 0.5	*a*_*R*_ = 0.25	*g* = 0.85	[18]
5	Sips	Rational	$q_{e}=\frac{q_{S}\;K_{S}C_{e}^{m_{S}}}{1+K_{S}C_{e}^{m_{S}}}$	/	*q*_*S*_ = 5.0	*K*_*S*_ = 0.1	*m*_*S*_ = 0.7	[19]

*Exponential.

**Table 2:** Types of weights.

Type of weights	Expression
Absolute weights	1
Poisson weights	1/*y*_*i*_^0.5^
Assumption of constant percentage error	1/*y*_*i*_
Instrumental weights	1/*sd*⁡*y*_*i*_

**Table 3:** Noise precision models.

No.	NPM	Expression	Type
1	H1	*σ* = 0.01	H*
2	H2	*σ* = 0.02	H
3	H3	*σ* = 0.05	H
4	H4	*σ* = 0.1	H
5	H5	*σ* = 0.2	H
6	H6	*σ* = 0.4	H
7	Lin	*σ* = 0.02 + 0.05*C*	Het**
8	Quad	*σ* = 0.02 + 0.02*C* + 0.0002*C* ^2^	Het
9	HS	*σ* = (0.02^2^+0.05^2^ *C* ^2^)^0.5^	Het
10	RSD5%	*σ* = 0.05*C*	Het
11	RSD10%	*σ* = 0.1*C*	Het

*H: homoscedastic; **Het: heteroscedastic.

**Table 4:** Definitions of error functions in cases when there are no replicated concentration measurements.

Name	Abbreviation	Domain	Expression
Theoretical orthogonal distance regression	TODR	Theoretical	$\overset{n}{\underset{i=1}{\sum }}\left[{\left(\frac{q_{ei,\exp}-q_{ei,\text{cal}}}{\sigma_{\varepsilon i}}\right)}^{2}+{\left(\frac{C_{ei,\exp}-C_{ei,\text{cal}}}{\sigma_{\delta i}^{e}}\right)}^{2}\right]$
Ordinary least squares	OLS	Experimental	$\overset{n}{\underset{i=1}{\sum }}{\left(q_{ei,\exp}-q_{ei,\text{cal}}\right)}^{2}$
Orthogonal distance regression	ODR	Experimental	$\overset{n}{\underset{i=1}{\sum }}\left[{\left(\frac{q_{ei,\exp}-q_{ei,\text{cal}}}{q_{ei,\exp}}\right)}^{2}+{\left(\frac{C_{ei,\exp}-C_{ei,\text{cal}}}{C_{ei,\exp}}\right)}^{2}\right]$
Marquardt's percent standard deviation	MPSD	Experimental	$\overset{n}{\underset{i=1}{\sum }}{\left(\frac{q_{ei,\exp}-q_{ei,\text{cal}}}{q_{ei,\exp}}\right)}^{2}$
Hybrid fractional error function	HYBRD	Experimental	$\overset{n}{\underset{i=1}{\sum }}\frac{{\left(q_{ei,\exp}-q_{ei,\text{cal}}\right)}^{2}}{q_{ei,\exp}}$

**Table 5:** Definitions of error functions for replicated measurements.

Name	Abbreviation	Domain	Expression
Experimental weighted orthogonal distance regression	E3WODR	Experimental	$\overset{n}{\underset{i=1}{\sum }}\left[{\left(\frac{{\overset{-}{q}}_{ei,\exp}-q_{ei,\text{cal}}}{\mathrm{sd}y_{i}}\right)}^{2}+{\left(\frac{{\overset{-}{C}}_{ei,\exp}-C_{ei,\text{cal}}}{{\mathrm{sd}x}_{i}}\right)}^{2}\right]$
Weighted least squares	WLS	Experimental	$\overset{n}{\underset{i=1}{\sum }}{\left(\frac{{\overset{-}{q}}_{ei,\exp}-q_{ei,\text{cal}}}{\mathrm{sd}y_{i}}\right)}^{2}$
Triplicate orthogonal distance regression	E3ODR	Experimental	$\overset{n}{\underset{i=1}{\sum }}\left[{\left(\frac{{\overset{-}{q}}_{ei,\exp}-q_{ei,\text{cal}}}{{\overset{-}{q}}_{ei,\exp}}\right)}^{2}+{\left(\frac{{\overset{-}{C}}_{ei,\exp}-C_{ei,\text{cal}}}{{\overset{-}{C}}_{ei,\exp}}\right)}^{2}\right]$

## Nomenclature

*a*_*R*_: Redlich-Peterson isotherm constant
*C*_*e*,true_: True equilibrium concentration
*C*_*e*,exp⁡_: Experimentally determined equilibrium concentration
${\overset{-}{C}}_{e,\exp}$: Mean of three experimentally determined equilibrium concentrations
*C*_*o*,true_: True adsorbate initial concentration
*C*_*o*,exp⁡_: Experimentally determined adsorbate initial concentration
*d*: Weights on the *x*-axis
*e*: Percentage error
*g*: The exponent in Redlich-Peterson isotherm
*i*: Indices for running number of data points
*j*: Running number of numerical experiments
*k*: Coefficient equal to *V*/*m*
*kf*: Number of converged fits in a simulation
*l*: Parameter ordinal number in the isotherm equation
*K*_*F*_: Freundlich adsorption constant related to adsorption capacity
*K*_*J*_: The exponent in Jovanovic isotherm
*K*_*L*_: The equilibrium adsorption constant in Langmuir equation
*K*_*R*_: The Redlich-Peterson isotherm parameter
*K*_*S*_: The Sips isotherm parameter
*m*: The weight of adsorbent
mE: Median percentage error
*m*_*S*_: The Sips model exponent
MARE: Mean absolute relative error
*n*: Number of observations
*n*_*F*_: Adsorption intensity in Freundlich isotherm
*p*: Number of parameters
*q*_*e*,exp⁡_: Experimentally determined adsorbent equilibrium loading
${\overset{-}{q}}_{e,\exp}$: Mean of three experimentally determined adsorbent equilibrium loadings
*q*_*e*,cal_: Equilibrium adsorbent loading calculated by isotherm equation
*q*_max⁡_: The Langmuir maximum adsorption capacity
*q*_*S*_: The Sips maximum adsorption capacity
*q*_*J*_: The Jovanovic maximum adsorption capacity
*sd**x*: Estimates of population standard deviation for independent variable
*sd**y*: Estimates of population standard deviation for dependent variable
*V*: The volume of adsorbate solution
*w*: Weights on the *y* axis
$\left({\hat{x}}_{i},{\hat{y}}_{i}\right)$: Data point on the fitted curve that is the closest to the observation (*x*_*i*_, *y*_*i*_).

### Greek Symbols

*ε*: Measurement error in dependent variable
*δ*: Measurement error in independent variable
*σ*_*ε*_: Population standard deviation of measurement error in dependent variable
*σ*_*δ*_: Population standard deviation of measurement error in independent variable
*σ*_*δ*^*o*^_: Population standard deviation of measurement error in initial concentration
*σ*_*δ*^*e*^_: Population standard deviation of measurement error in equilibrium concentration
***θ***: Vector of true parameters
$\hat{\theta }$: Vector of estimated parameters
**φ**: Vector of distances in *x* direction between the observations and true model
$\hat{\varphi }$: Vector of distances in *x* direction between the observations and model function with estimated parameters
*ψ*: Vertical distance between observation and model function.
