# Supplementary material for: A New Approach in Regression Analysis for Modeling Adsorption Isotherms
Source: ScientificWorldJournal. 2014 Jan 30;2014:930879. doi: 10.1155/2014/930879 (PMC3929603; doi:10.1155/2014/930879)
Supplement: Supplementary file 1 — Graphical representation of the results obtained in case when adsorption data is collected in repeated experiments is presented in Supplementary material. Properties of applied methods are compared in terms of median of percentage error and mean absolute relative error for the isotherm parameter determination. [file 930879.f1.pdf]

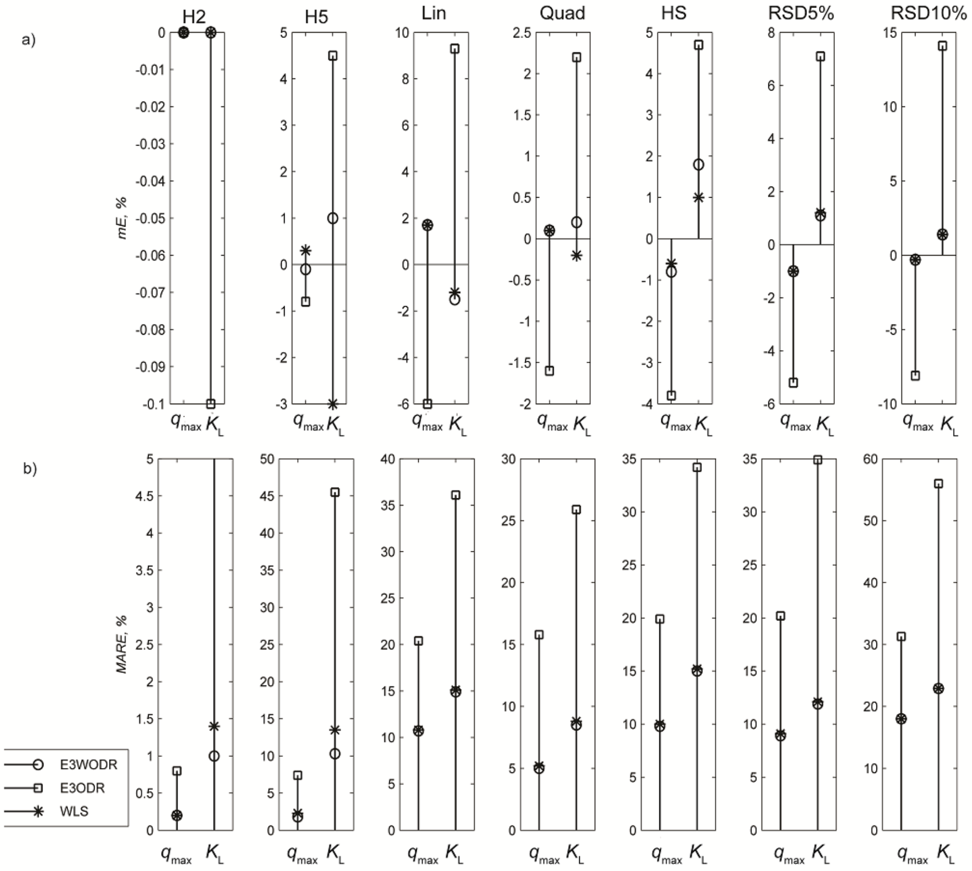

FIGURE A.1: Properties of different modeling approaches for determination of parameters in Langmuir isotherm (experiments performed three times), a) *mE* , b*) MARE*.

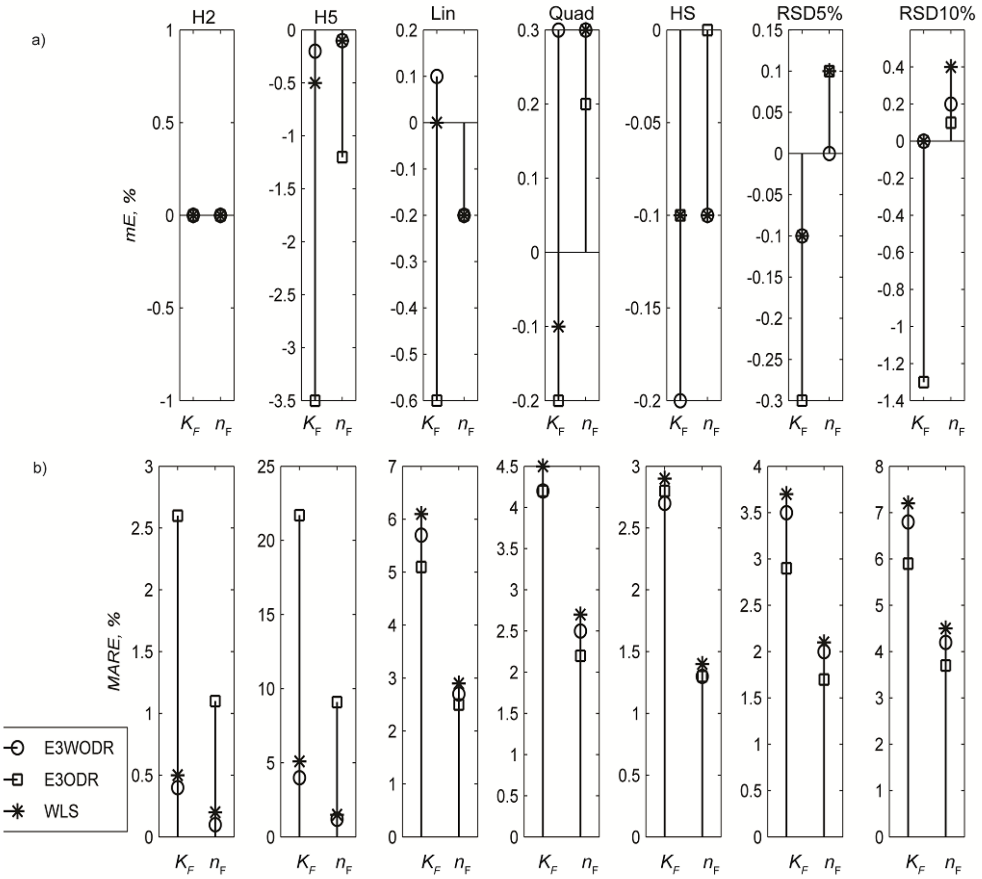

FIGURE A.2: Properties of different modeling approaches for determination of parameters in Freundlich isotherm (experiments performed three times), a) *mE* , b*) MARE*

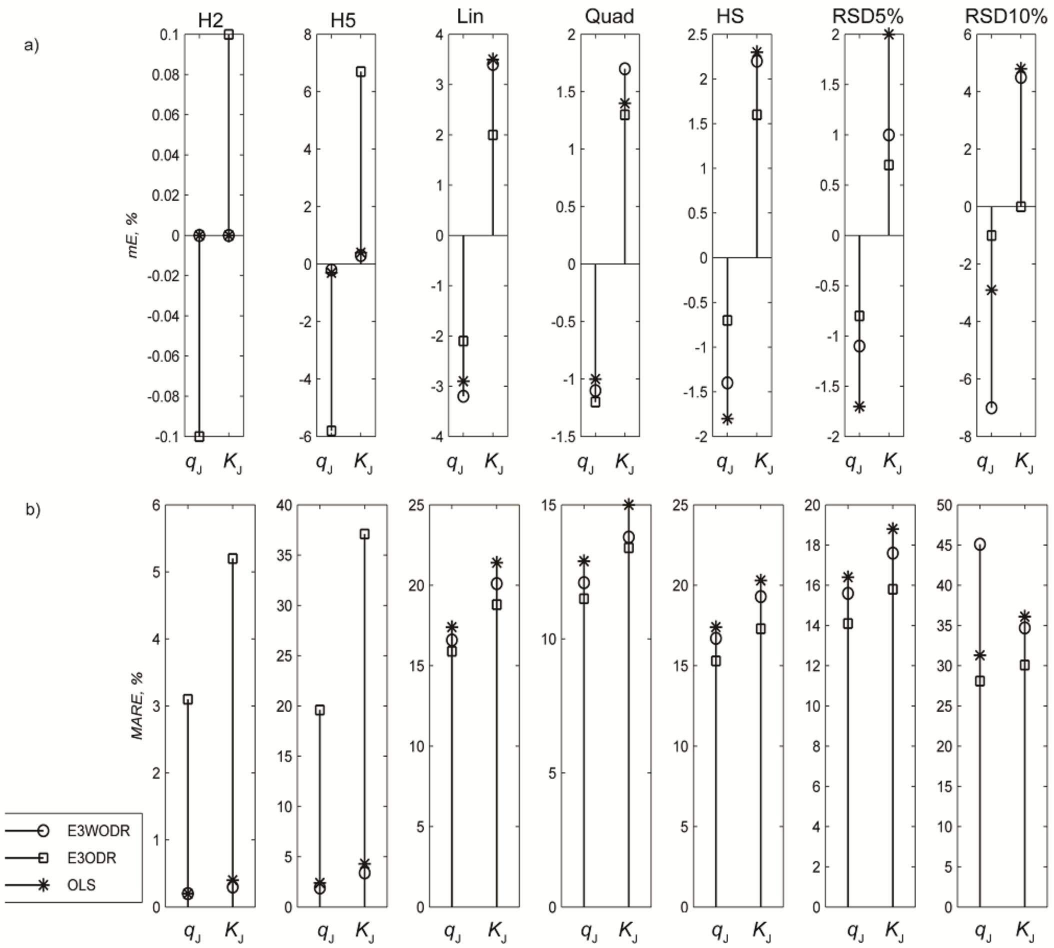

FIGURE A.3: Properties of different modeling approaches for determination of parameters in Jovanovic isotherm (experiments performed three times), a) *mE* , b*) MARE*

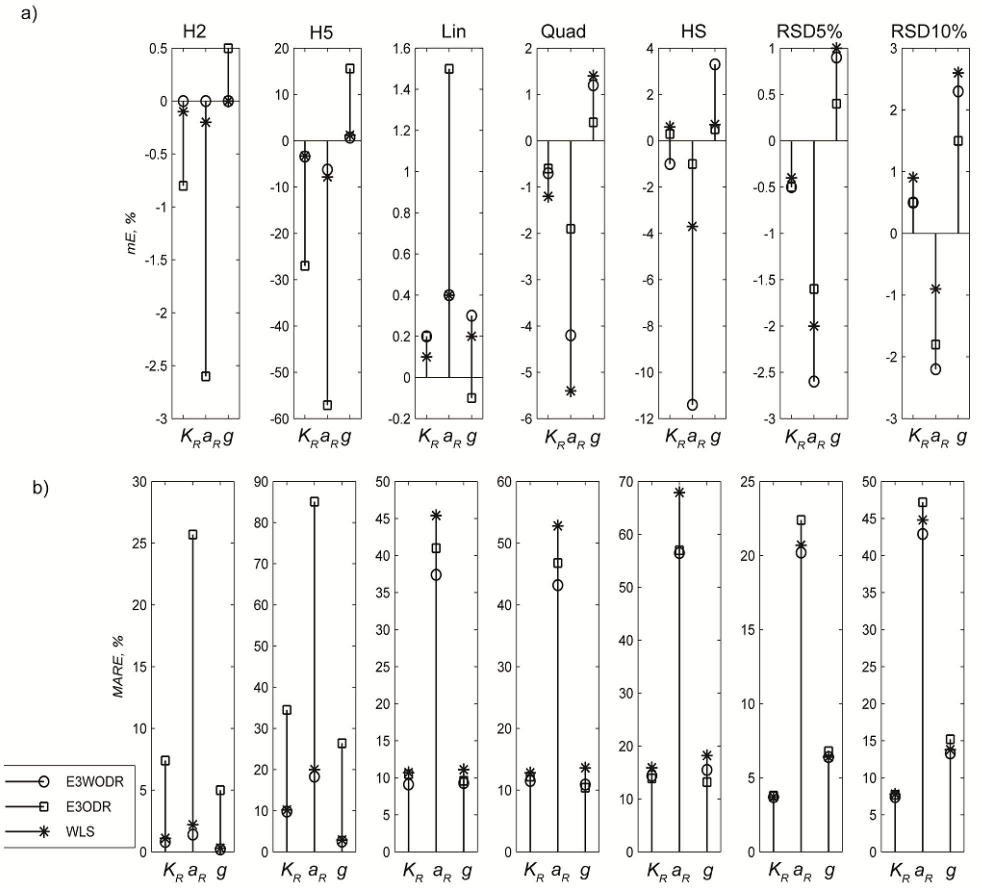

FIGURE A.4: Properties of different modeling approaches for determination of parameters in Redlich-Peterson isotherm (experiments performed three times), a) *mE* , b*) MARE*

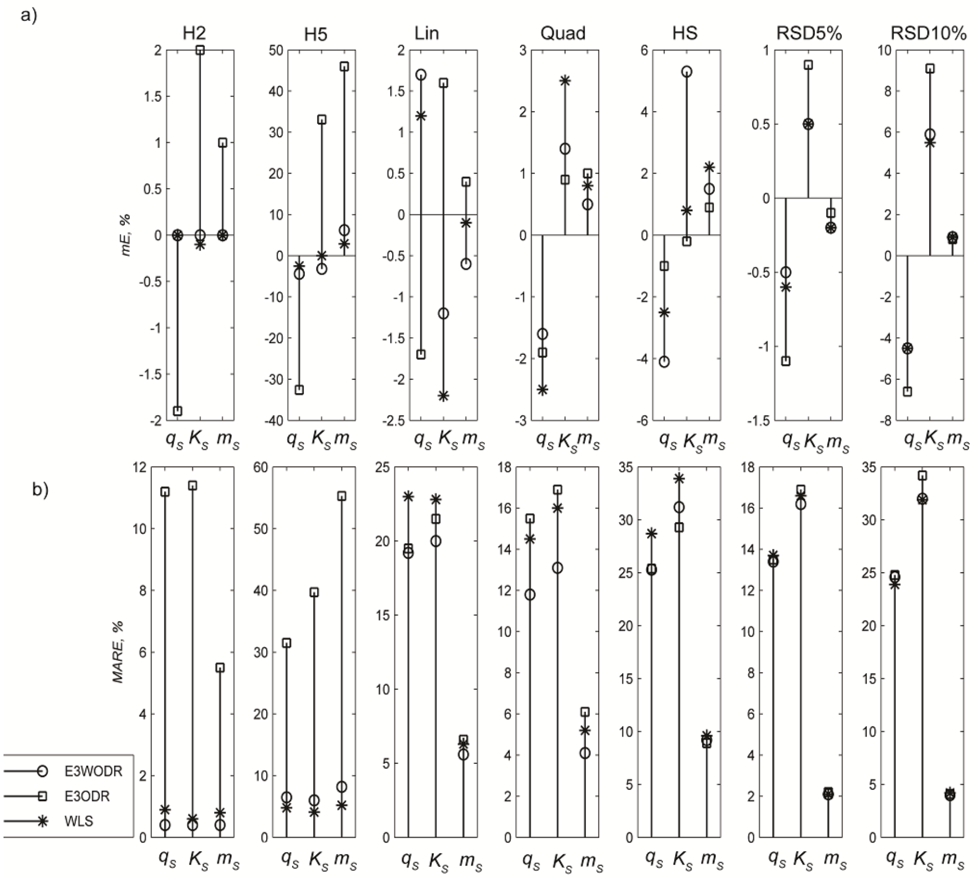

FIGURE A.5: Properties of different modeling approaches for determination of parameters in Sips isotherm (experiments performed three times), a) *mE* , b*) MARE*
